# AgNiCoCr Nanoparticles: Exploring the Morphological Shift from Elementally Segregated to High Entropy Structures

**DOI:** 10.1021/acsanm.6c02061

**Published:** 2026-08-15

**Authors:** Soumya Mandal, Avik Mahata, Annaliese Colwell, Vikas Reddy Paduri, Nagarajan Anna Ramesh Babu, Ritesh Sachan

**Affiliations:** † Department of Mechanical and Aerospace Engineering, 7618 Oklahoma State University, Stillwater, Oklahoma 74078, United States; ‡ Department of Mechanical & Electrical Engineering, 6055 Merrimack College, North Andover, Massachusetts 01845, United States; § Department of Materials Science and Engineering, 7712 University of Connecticut, Storrs, Connecticut 06269, United States

**Keywords:** nanosecond laser-induced dewetting, pulsed laser irradiation, high entropy alloy nanoparticles, AgNiCoCr, transmission electron microscopy

## Abstract

Controlling elemental mixing in nanoparticles composed of immiscible elements remains a central challenge in high-entropy materials design. Here, we demonstrate a kinetic pathway that enables a transition from elementally segregated to high-entropy AgNiCoCr nanoparticles using nanosecond laser-induced dewetting of metallic thin films. During this nonequilibrium process, nanoparticles form through a sequence of morphological transformations and ultimately evolve into near-spherical structures with thickness-dependent sizes. In the chosen model system, Ag is thermodynamically immiscible with Ni and Co, whereas Ni, Co, and Cr are mutually miscible at equiatomic compositions. By varying the thickness and configuration of the metallic thin-film layers, the liquid-phase lifetime during laser irradiation is systematically tuned, thereby regulating mass transport and solidification dynamics. Ultrathin film stacks produce smaller nanoparticles (up to ∼40 nm) that rapidly solidify on nanosecond time scales, kinetically trapping metal atoms into a chemically disordered high-entropy phase. In contrast, thicker films remain molten for longer durations during dewetting, leading to the formation of larger nanoparticles with pronounced elemental segregation into AgNiCoCr core–shell or Janus structures. Supported by atomistic simulations, this work demonstrates that solidification kinetics, rather than thermodynamic immiscibility, governs chemical order in laser-processed nanoparticles, providing a versatile strategy for engineering high-entropy nanoparticles from immiscible elements.

## Graphical abstract

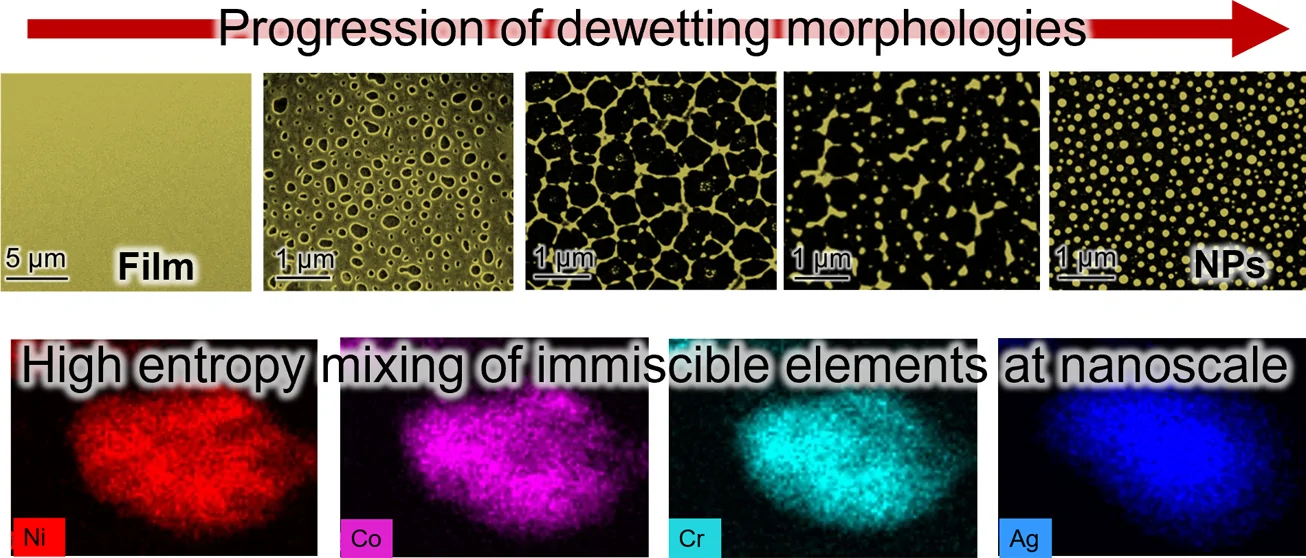

## 1 Introduction

Multimetallic nanoparticles (NPs) have emerged as a versatile class of functional nanomaterials due to their tunable optical, catalytic, magnetic, and electronic properties, which arise from the synergistic interplay of size, shape, and chemical composition. In particular, NPs composed of multiple principal elements enable access to property spaces that are unattainable in conventional monometallic or bimetallic systems, making them attractive for applications ranging from plasmonics and sensing to catalysis and energy conversion. However, controlling elemental mixing and distribution within multicomponent NPs remains a fundamental challenge, especially when constituent elements exhibit strong thermodynamic immiscibility.^1–5^

High-entropy alloy (HEA) NPs, defined by the principal compositions of elements and high configurational entropy, offer a promising route to stabilize chemically disordered states in systems composed of mutually fully or partially miscible elements.^6,7^ While HEAs are well established in bulk and thin-film forms, extending the high-entropy concept to NPs is more recent and introduces additional complexity. Based on the established criteria for defining high-entropy alloying in the NPs, it requires random and homogeneous distribution of the constituent elements within a single, solid-solution crystal phase, rather than forming segregated clusters.^8^ At the nanoscale, thermodynamics, enhanced surface-to-volume ratios, reduced diffusion lengths, and surface energy minimization strongly favor elemental segregation, often resulting in core–shell or Janus morphologies rather than homogeneous solid solutions and making HEA NPs formation more challenging.^9–11^ Several bottom-up approaches, including chemical coreduction, seeded growth, photochemical synthesis, and top-down approaches, such as carbothermal shock synthesis and laser ablation in liquids, have been explored to fabricate multielement NPs.^10,12,13^ Although these methods can produce colloidal NPs with controlled average compositions, they often suffer from limited control over particle size dispersion, spatial arrangement, and elemental homogeneity, particularly for immiscible systems. Moreover, many photonic, catalytic, and device-oriented applications require NP arrays directly supported on substrates, which further constrains the applicability of solution-based techniques. These limitations highlight the need for alternative synthesis strategies that operate far from equilibrium and allow independent control over NP size, spacing, and chemical order.

Nanosecond laser-induced dewetting (NLiD) of ultrathin metallic films provides an effective route for fabricating ordered NP arrays via a top-down, self-organization process and enables access to a variety of metallic systems. In NLiD, nanosecond laser irradiation melts a continuous thin film, which subsequently undergoes hydrodynamic instabilities driven by surface tension, thermocapillary forces, and interfacial energy minimization. The molten film ruptures into holes, polygonal networks, or bicontinuous structures, which further evolve into isolated, near-spherical NPs upon repeated melting and resolidification. This approach has been extensively studied for single and bimetallic films, demonstrating excellent control over NP size and spacing through film thickness, substrate properties, and laser parameters.^14–19^ However, extending NLiD to a larger space of multicomponent systems containing immiscible elements raises several questions regarding elemental transport, mixing, and segregation during ultrafast melting and quenching. Till date, in the view of NPs consisting of multiprincipal element alloys (MPEAs) or high entropy alloys (HEAs), there are only two reported works in the best of our knowledge including one from our research group on NiCoCr^20^ and the other by Ghebretnsae et al.^21^ on AgCuPdPtAu. In addition, synthesis of HEA polyhedral NPs has been reported using liquid metal dewetting. In pulsed laser-driven dewetting, the NP formation process occurs on nanosecond time scales, during which liquid-state diffusion competes directly with rapid solidification.^22^ This extreme nonequilibrium environment provides an opportunity to kinetically suppress phase separation and trap chemically disordered states in a crystalline lattice structure that would be unstable under equilibrium conditions. Yet, the extent to which such kinetic trapping can overcome thermodynamic immiscibility, and how it depends on film thickness, layer configuration, and molten lifetime, remains poorly understood, particularly for systems with strong chemical driving forces for segregation.

In this work, we address this challenge using AgNiCoCr as a model multicomponent system. Ag is thermodynamically immiscible with Ni and Co, while Ni, Co, and Cr form a stable solid solution at equiatomic compositions.^23,24^ This combination provides a stringent test case for evaluating whether nonequilibrium laser processing can stabilize HEA NPs against segregation. By systematically varying thin-film thickness (∼5–20 nm) and deposition configuration (NiCoCr/Ag, Ag/NiCoCr, and codeposited films), we aim to control the liquid-phase lifetime during nanosecond laser irradiation and directly probe its impact on NP morphology, size, and elemental distribution. We hypothesize that ultrathin films solidify within a few nanoseconds, kinetically trapping a chemically disordered, high-entropy state, whereas thicker films remain molten long enough to enable Ag surface segregation and the formation of core–shell or Janus morphologies. Through a combination of scanning (SEM) and transmission electron microscopy (TEM), thermal transport modeling, and large-scale molecular dynamics (MD) simulations, we test the hypothesis and develop our understanding on a transition from elementally segregated NPs to homogeneously mixed high-entropy AgNiCoCr NPs as a function of film thickness. Although the NP dimensions accessible by MD simulations are significantly smaller than those observed experimentally because of computational limitations, the simulations are intended to capture the atomistic mechanisms governing rapid melting, diffusion, elemental segregation, and solidification rather than to reproduce the experimental particle sizes quantitatively. These local atomic processes are governed primarily by interatomic energetics and diffusion kinetics, which are expected to remain qualitatively applicable across the experimentally investigated size range. Therefore, the MD simulations provide mechanistic insight into the origin of the thickness-dependent transition from chemically homogeneous to segregated nanoparticles observed experimentally. By explicitly linking film thickness to molten lifetime, diffusion length, and solidification kinetics, this study establishes a quantitative framework for engineering chemical order in multimetallic NPs via nonequilibrium laser processing. Overall, this work demonstrates that solidification kinetics, rather than thermodynamic miscibility alone, governs elemental distribution in NLiD-driven NPs. The insights gained here provide a general strategy for designing high-entropy NPs from immiscible elements and open new pathways for fabricating compositionally complex nanostructures for plasmonic, catalytic, and energy-related applications.^25,26^

## 2 Experimental Methodology

### 2.1 Pulsed Laser-Induced Dewetting

AgNiCoCr thin films were deposited on two substrates (SiO_2_/Si and carbon) utilizing the pulsed laser deposition (PLD) technique. In the first, ultrathin films of NiCoCr and Ag (thickness ranging between 2 and 10 nm) were deposited on commercially available, optical quality Si wafers with a ∼300 nm thick thermally grown oxide layer. In the second case, carbon films (∼20 nm) were first deposited on commercially available V-2 quality freshly cleaved mica, followed by NiCoCr and Ag thin films where carbon acted as a final electron-transparent substrate.^27^ In both cases, the deposition is carried out in a high vacuum (10^–7^ Torr) environment. High purity (>99.99%) circular (2.54 cm diameter × 0.5 cm thickness) ablation target of NiCoCr (having an equimolar composition of Ni, Co, and Cr) and Ag was used for PLD. The NiCoCr and Ag target utilized in this experiment was purchased from Sophisticated Alloys, Inc. which was prepared using the vacuum arc melting technique. Before performing the experiments, the PLD targets and the SiO_2_/Si wafers were cleaned by sonication using successive baths of acetone, methanol, isopropyl alcohol, and DI water to effectively eliminate surface impurities. A nanosecond pulsed KrF laser having a wavelength of 248 nm and a pulse width of 25 ns was used to ablate the PLD target.^28,29^ Three types of NiCoCr and Ag film configurations (NiCoCr/Ag, Ag/NiCoCr, and codeposited) were deposited on substrates (SiO_2_/Si and carbon) while varying film thicknesses between ∼5 nm, ∼10 nm, and ∼20 nm). For both NiCoCr/Ag and Ag/NiCoCr configurations, the thicknesses of each layer were maintained evenly. For example, to achieve the total thickness of ∼5 nm in NiCoCr/Ag configuration, ∼2.5 nm of NiCoCr was deposited on top of ∼2.5 nm of Ag. A similar method was followed for fabricating the Ag/NiCoCr films configuration of different thicknesses. A sectional ablation target of NiCoCr and Ag was used for depositing the codeposited films of different thicknesses. By adjusting the laser pulses, the deposition process was carried out at a laser fluence of ∼1.5 mJ.cm^–2^ to deposit continuous films of NiCoCr and Ag. The applied laser fluence was sufficient to ablate the metal targets and initiate the deposition process. During the deposition, the optimum distance between the substrates (SiO_2_/Si and carbon) and the ablation targets were maintained at ∼4 cm. A larger distance than ∼4 cm slowed down the deposition rate of the constituent elements, whereas the shorter distance allowed the larger particulates to deposit on the thin films. As a result, the ablated Ag, Ni, Co, and Cr were deposited on the SiO_2_/Si and carbon substrate as a continuous bilayer and codeposited films. After deposition, the PLD vacuum chamber was gently filled with argon gas until the internal pressure equaled the atmosphere, and the as-deposited thin films were carefully removed from the PLD chamber. Atomic force microscopy (AFM) was used to measure the thickness and roughness of the as-deposited thin films. The roughness of thin films was concluded to be 0.1 ± 0.03 nm (upper limit) for the average root-mean-square (RMS) roughness. Interestingly, no significant change in the surface roughness of the as-deposited films was observed with the change in film configuration and thickness. The as-deposited thin films were irradiated using the same nanosecond pulsed laser with a repetition rate of 25 Hz in ambient environment. Due to the Gaussian energy distribution profile of the laser beam, an aperture was utilized to keep the beam spot at ∼3 mm to reduce the inhomogeneity of the laser energy for the irradiation experiments. The portion of the laser beam that was allowed through the aperture had a uniform energy distribution and a laser fluence that was more than the minimum threshold required to completely melt the deposited thin films in the entire region. The pulsed laser irradiation was carried out at a laser fluence of 200 mJ.cm^–2^, which was sufficient to melt the thin films of different thicknesses and initiate the dewetting phenomenon.

### 2.2 Electron Microscopy Characterization

The FEI Quanta 600 scanning electron microscopy (SEM) operating at 20 kV was used to characterize the dewetting morphologies together with the formation of AgNiCoCr NPs. The average size of the NPs was estimated from the size distribution histogram generated from the SEM images. The fast Fourier transforms (FFTs) of the SEM images were used to examine the average nearest neighbor spacing of the NPs.

A JEOL JEM-2100F transmission electron microscopy (TEM) operating at 200 kV was used to collect high-resolution data of AgNiCoCr NPs fabricated via dewetting. The elemental and compositional distribution of the constituent elements (Ag, Ni, Co, and Cr) in the NPs were also investigated using EDS/TEM.

TEM samples were prepared by using the floating-off technique using the samples deposited on carbon film. The preparation of the TEM samples using the floating-off technique involves two stages. The irradiated samples forming the NPs on carbon/mica were inserted into water which causes separation of carbon film with mica leading to NPs forming on a continuous carbon film and floating on water surface. AgNiCoCr NPs was successfully collected on Cu and Au TEM grids. The TEM grids were then heated at 60 °C for 2 h to dry it and make it ready for the TEM investigation.^27^

### 2.3 Thermal Transport Simulations

The nanoscale thermal transport simulation of AgNiCoCr thin films deposited on a SiO_2_/Si substrate was performed with a 2D finite element model utilizing the time-dependent heat transfer in solids in COMSOL Multiphysics.^30,31^ For the thermal modeling of thin films, the heat transfer mode and transient analysis in the conduction type of heat transfer were taken into account. In the simulation, a single laser pulse was applied on the top surface of thin films, and the rest of the surfaces were considered thermally insulated. The ambient temperature was set at 293 K. For the improved accuracy of the thin film simulation, mesh elements with an exceptionally finer distribution and a maximum size of 2 nm were utilized. For the thermal profiles, a time range of 0 to 100 ns with a 0.01 ns step size was chosen.

### 2.4 Molecular Dynamics Simulation

We performed classical molecular dynamics in LAMMPS.^32^ The simulation box (250 × 250 × 20 Å; nonperiodic (shrink-wrapped) in x and y, periodic in z) contained a multilayer stack centered in *Z* direction: crystalline Si (8 Å), amorphous SiO_2_ (8 Å), an Ag interlayer, and an fcc NiCoCr film. Ag and NiCoCr layer thicknesses were scanned from 1 to 5 Å in 0.5 Å steps; because a 1 Å Ag slab can fail to populate a continuous layer while 5 Å is unrealistically thick for our purpose, analysis focuses on 2, 3, and 4 Å. Metals were modeled with an EAM/alloy description (Co–Ni–Cr–Ag),^33^ the substrate/oxide with a Tersoff Si–O potential, and metal–substrate cross interactions with 12–6 Lennard-Jones terms derived from UFF^34^ and converted to LAMMPS metal units; neighbor lists used a 2.0 Å bin size. Initial velocities were assigned at 300 K. The thermal cycle mimicked laser-induced dewetting and quench used experimentally, producing NP droplets after resolidification; thickness is therefore the primary control parameter connecting melt lifetime and segregation tendency. Three nominal peak temperatures (2000 K, 2500 K and 3000 K). We also simulated NiCoCr on top of Ag and Ag on top of NiCoCr alloy. A total of three temperatures, two stacking orders, and 10 thickness values yielded 60 simulations, and we repeated each simulation three times for statistical differences (180 simulations in total).

Atomic configurations were postprocessed with a custom Python/OVITO^35^ workflow on the final (solid) frame. We computed radial composition profiles *c*_i_(*r*) about the NP center of mass and defined the core–shell index for Ag, CSI_Ag_ = *c*_surf_ – *c*_core_, with core rR<0.5 and surface 0.8<rR≤1 . Surface enrichment was quantified by EAg=coutxAg (outer shell thickness 0.2 nm), and species positions by the normalized mean radius ⟨*r*_i_⟩/*R*. Short-range order (Warren–Cowley)^36^ was evaluated as αij=1−pijcij in core and surface regions using a first-shell cutoff taken at the first minimum of the partial *g*_*ij*_(*r*) (≈3.3 Å; results are insensitive within ±0.1 Å). Finally, we computed a dimensionless segregation number, Σ≡DrelτliqL2 (formally the mass-diffusion Fourier number), where *L* is final particle radius, τ_liq_ the molten lifetime from the MD thermal history, and *D*_rel_ the liquid interdiffusion coefficient from the long-time Einstein relation $D_{rel}=\left(\frac{1}{6}\right)$ d­(MSD)/d*t* in a separate equilibrium-liquid simulation. Together, these metrics link thickness-controlled quench kinetics to Ag surface enrichment and Ni/Co/Cr core formation.

In addition to the static segregation number *S* derived from composition metrics, we also computed a dynamic, dimensionless segregation number Σ that captures the competition between melt lifetime and atomic diffusion. Specifically, the molten lifetime (τ_liq_) was extracted from time-resolved MD trajectories by tracking the time taken to form the first NP from the bicontinuous thin film. The average particle radius, *L*, was obtained OVITO by averaging several NPs at the end of the simulation. Together with the relative liquid interdiffusion coefficient *D*_rel_, obtained from the slope of the long-time mean-squared displacement in separate equilibrium liquid simulations, these values were used to calculate Σ=DrelτliqL2 . This definition is formally equivalent to a Fourier number for mass diffusion and serves as a universal criterion: Σ < 1 predicts a kinetically trapped HEA-like state, while Σ > 1 predicts segregation into an Ag-rich shell and NiCoCr-rich core. This dimensionless segregation number Σ is analogous to Fourier-type criteria used in rapid solidification^37^ and to scaling analyses of solid-state dewetting in binary alloys,^38^ where the competition between diffusion time scales and morphological evolution governs whether a homogeneous alloy or a segregated core–shell results. By compiling Σ across 2, 3, and 4 Å bilayers at 2000–3000 K and both stacking orders, we generated a Σ-map that places each simulation outcome relative to the Σ = 1 transition boundary.

## 3 Results and Discussion

Figure 1 presents plan-view SEM images illustrating the evolution of nanoscale morphologies during laser-induced dewetting of thin films with different stacking configurations, namely NiCoCr/Ag, Ag/NiCoCr, and codeposited NiCoCr–Ag. The as-deposited films with total thicknesses of approximately 5, 10, and 20 nm were irradiated at a laser fluence of 200 mJ cm^–2^ with an increasing number of pulses. In all cases, progressive irradiation leads to the formation of well-separated AgNiCoCr NPs. Interestingly, although the overall film thickness is identical for the different configurations, the average size of the resulting NPs varies depending on the interfacial perturbation frequency and following dewetting pathway. The average NP diameter ranges from 40 ± 8 nm to 310 ± 30 nm as the total film thickness increases from ∼5 nm to ∼20 nm. During laser irradiation, the rapid melting and subsequent resolidification lead to the formation of droplets that quickly quench into near-spherical NPs, minimizing the overall surface energy of the system.^39^ The total surface energy is governed by surface tension at the film–air and film–substrate interfaces, as well as intermolecular interactions between the constituent layers. The melting-solidification cycle associated with each laser pulse occurs within approximately 100 ns, which is significantly shorter than the interval between successive pulses (40 ms at a repetition rate of 25 Hz). Consequently, the NPs solidify into energetically stable geometries after each pulse, resulting in no significant change in particle shape during subsequent irradiation cycles. Once dewetting is initiated by laser irradiation, thin films undergo a sequence of morphological transitions that ultimately lead to NP formation. Typically, morphology evolves through either a hole-to-polygonal pathway or a bicontinuous pathway before the final NP state is reached. The breakup of the intermediate polygonal or bicontinuous structures into discrete NPs occurs through a Rayleigh-like instability, where thermocapillary-driven mass transport promotes the redistribution of molten material and the eventual formation of isolated NPs.^14,40^ Variations in NP size distributions are attributed to differences in capillary velocities of the molten metals, which depend on the interfacial tension and viscosity of the liquid phase.^41^

**Figure 1:**
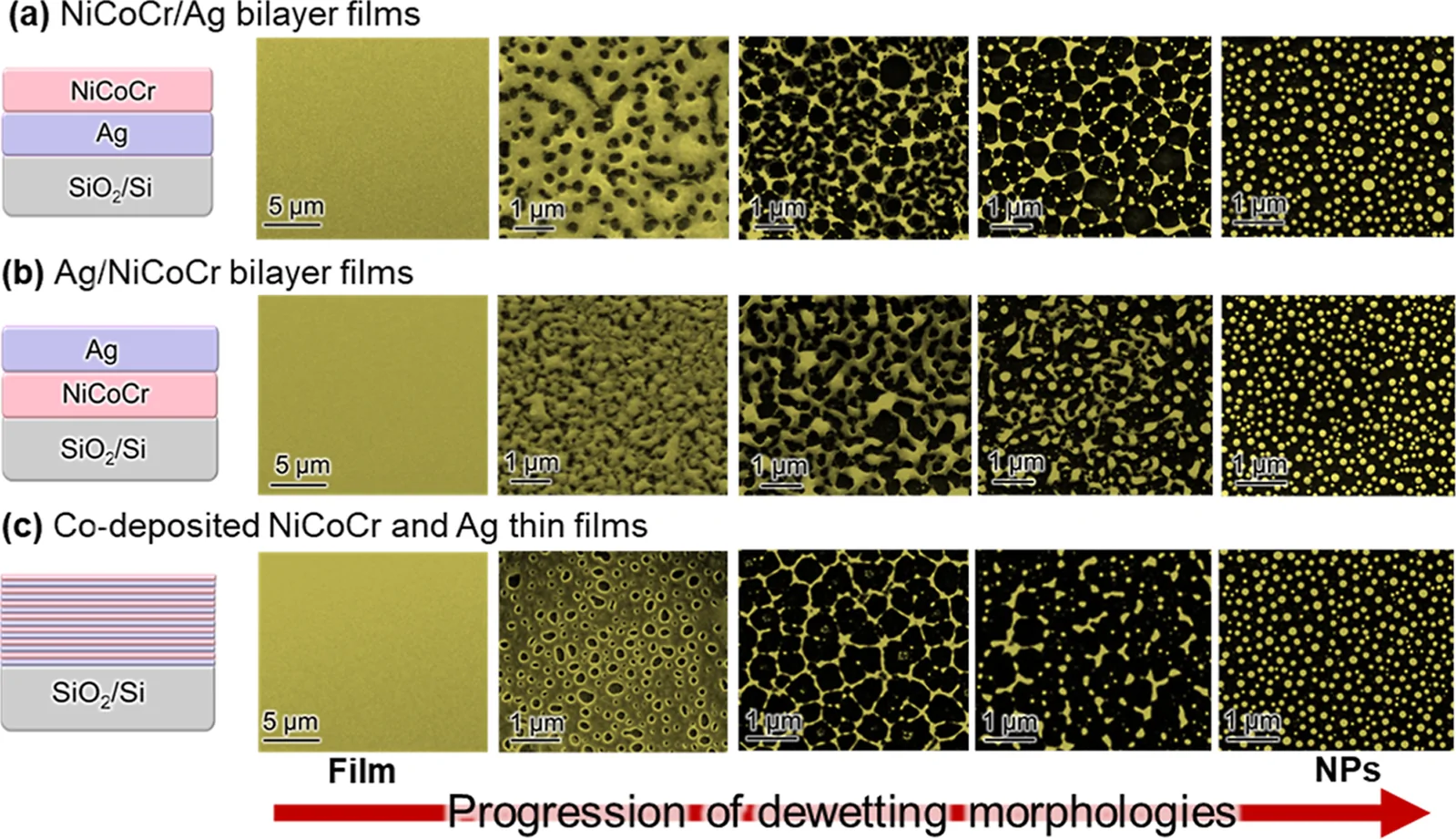
Plan-view SEM images showing the evolution of nanoscale morphologies during laser-induced dewetting of thin films (∼10 nm total thickness): (a) NiCoCr/Ag bilayers, where the morphology evolves from holes to polygonal networks and finally to NPs with increasing laser pulses; (b) Ag/NiCoCr bilayers, where the dewetting pathway proceeds through bicontinuous and spaghetti-like structures before forming NPs; and (c) codeposited NiCoCr–Ag films, where both polygonal and bicontinuous intermediate morphologies appear prior to NP formation (only the pathway via polygonal structures is shown in the figure). In all cases, continued laser irradiation drives mass transport in the molten state, leading to arrays of well-separated AgNiCoCr NPs.

For the NiCoCr/Ag bilayer configuration as shown in Figure 1a, the early stage of dewetting is characterized by the nucleation of nearly uniform holes as the ultrathin film destabilizes upon melting. With increasing laser pulses, these holes expand and coalesce with neighboring holes, producing polygonal network structures. Continued irradiation enhances melt-stage mass transport and capillary-driven material redistribution, causing the polygonal structures to break up and eventually form near-spherical AgNiCoCr NPs. In contrast, the Ag/NiCoCr bilayer configuration shown in Figure 1b follows a different pathway in which the intermediate morphology consists of bicontinuous structures rather than polygonal networks. At the early stages of dewetting, the film evolves into a bicontinuous pattern that subsequently transforms into spaghetti-like nanostructures with increasing laser pulses. Further irradiation drives the breakup of these elongated features into discrete AgNiCoCr NPs.

Unlike the bilayer configurations, codeposited NiCoCr–Ag films exhibit a combination of both mechanisms during the dewetting process. As shown in Figure 1c, the films initially develop polygonal network morphologies leading to NP formation, while in other regions the evolution proceeds through bicontinuous structures (see Figure S1). The coexistence of both intermediate morphologies in the codeposited films likely arises from the more complex scenario of perturbed interfaces within these films, which can also be viewed as ultrathin multilayer structures. Despite the distinct intermediate morphologies, both pathways ultimately yield NPs with similar average diameters (∼90 ± 15 nm), indicating that the final particle size is primarily governed by the initial film thickness rather than the specific dewetting pathway in the codeposited films.

The evolution of dewetting morphologies in the different film configurations is primarily governed by the thickness and identity of the top layer in the bilayer thin films. For instance, when NiCoCr forms the top layer, the formation of AgNiCoCr NPs proceeds through hole nucleation followed by polygonal network structures, which closely resemble the dewetting pathway observed in single-layer NiCoCr thin films. In contrast, when Ag is deposited as the top layer, the intermediate morphologies predominantly consist of bicontinuous structures, which persist until the Ag layer thickness reaches approximately ∼11 nm. For thicker Ag layers (≥∼11 nm), the intermediate morphologies transition to hole and polygonal structures, consistent with the dewetting behavior previously reported for single-layer Ag films.

While Figure 1 describes the morphological evolution during laser-induced dewetting, Figure 2 demonstrates that the resulting NP diameter and nearest-neighbor spacing scale with the initial film thickness, reflecting the intrinsic film thickness-dependent melt-state perturbation selection associated with spinodal thin-film instability. Figure 2a–c presents representative plan-view SEM images of AgNiCoCr NPs fabricated via nonequilibrium NLiD of thin films with total thicknesses of ∼5 nm, ∼10 nm, and ∼20 nm for the NiCoCr/Ag, Ag/NiCoCr, and codeposited NiCoCr–Ag configurations. Multiple SEM images were analyzed for each sample to obtain statistically reliable values of the average NP diameter and nearest-neighbor (NN) spacing. The results show that both the average NP diameter and NN spacing increase systematically with increasing initial film thickness, consistent with the scaling behavior expected for thin-film dewetting. For films with a total thickness of ∼5 nm, the average NP diameters are 50 ± 8 nm and 40 ± 8 nm for the NiCoCr/Ag and Ag/NiCoCr configurations, respectively, while the codeposited films produce slightly smaller NPs with an average diameter of 35 ± 8 nm. When the initial film thickness increases to ∼10 nm, the NP diameter correspondingly increases and exhibits a unimodal size distribution for all configurations. The average NP diameter is 115 ± 15 nm for the NiCoCr/Ag films and 100 ± 15 nm for the Ag/NiCoCr configuration, whereas the codeposited films yield NPs with an average diameter of ∼90 ± 15 nm. Further increasing the film thickness to ∼20 nm results in significantly larger NPs. The average NP diameters reach 310 ± 30 nm for NiCoCr/Ag films and 270 ± 30 nm for Ag/NiCoCr films, while the codeposited configuration produces NPs with an average diameter of ∼250 ± 30 nm. The nearest-neighbor spacing between NPs was analyzed using radial distribution functions (RDFs) obtained from the FFT patterns of the SEM images. The diffuse ring patterns observed in the FFTs indicate the presence of short-range ordering within the NP arrays. The peak position in the frequency space corresponds to the characteristic NN spacing between NPs. For films with an initial thickness of ∼5 nm, the average NN spacing is approximately 190, 150, and 130 nm for the NiCoCr/Ag, Ag/NiCoCr, and codeposited configurations, respectively. As the film thickness increases to ∼20 nm, the NN spacing correspondingly increases to approximately 900, 800, and 700 nm, respectively. These results are further demonstrated in Figure 3 showing an increase in particle size and NN-spacing with increasing bilayer or codeposited film thickness. Interestingly, the NPs fabricated from NiCoCr/Ag arrangements are exhibiting bigger diameters and larger nearest neighboring spacing over the Ag/NiCoCr arrangements regardless of initial film thickness, which is primarily associated with thermal conductivity and cohesive energy of NiCoCr and Ag. NiCoCr (15 W m^–1^ K^–1^) has substantially lower thermal conductivity than that of pure Ag (429 W m^–1^ K^–1^).^42^ Lower thermal conductivity slows down the heat transmission, which in turn causes cooling to proceed more slowly. As a result, the NiCoCr will remain in the liquid phase for an extended period, speeding up surface diffusion. At temperatures well above their melting point, particularly in the liquid phase, atoms exhibit higher mobility, thus accelerating surface diffusion. In addition, cohesive energy, which is the intermolecular force between the atoms, is primarily responsible for the mobility of metals in their liquid phase. NiCoCr (5.07 eV/atom) has a larger cohesive energy than pure Ag (2.95 eV/atom), which enables the likelihood that NiCoCr will diffuse over longer distances and accumulate to form larger NPs.^43^ Interestingly, NPs fabricated from codeposited thin films are the smallest in diameter and distributed closely with each other. This phenomenon can be explained based on the solidification rate, which is associated with the initial film thickness of NiCoCr and Ag. In the case of the codeposited configuration, the films are considerably thinner than the bilayer films (NiCoCr/Ag and Ag/NiCoCr), which accelerates the rate of heat transfer and thus in turn increases the solidification rate. The entire solidification process occurs so fast that the constituent elements (Ag, Ni, Co, and Cr) of the solid solution do not get adequate time to travel a larger distance for making bigger NPs. In essence, the Ag and NiCoCr will not be in their liquid phase for long enough to speed up the surface diffusion, which ended up forming smaller NPs with narrower nearest neighbor spacing.

**Figure 2:**
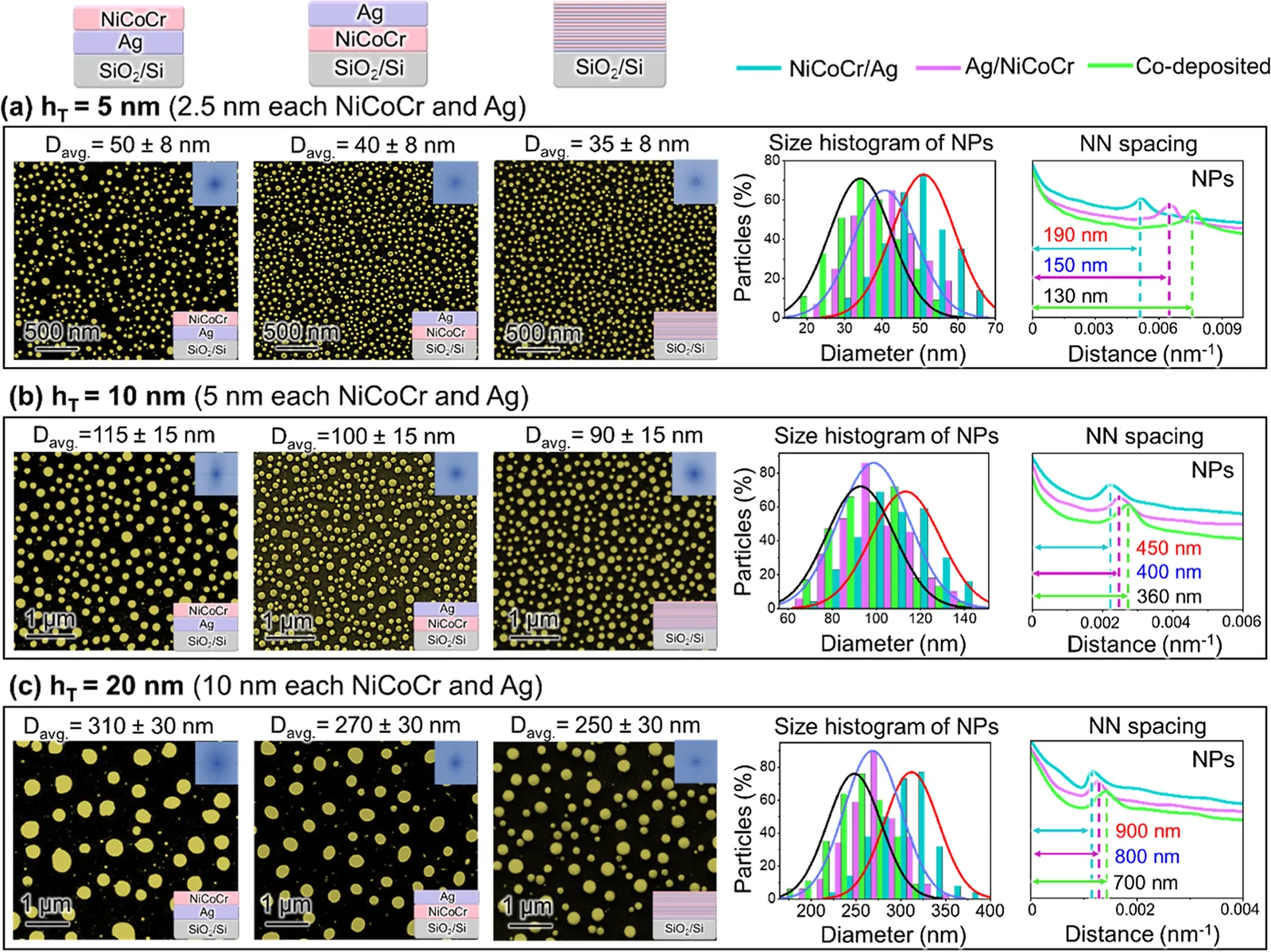
Plan view SEM images showing the AgNiCoCr NPs fabricated from thin films with total thickness of (a) ∼5 nm thin films, (b) ∼10 nm thin films, (c) ∼20 nm thin films having NiCoCr/Ag, Ag/NiCoCr and codeposited configurations respectively, together with their size histogram and nearest neighbor (NN) spacing. The fast Fourier transform (FFT) images in the inset show the spatial short-range ordering of NP arrays. The peak position in the NN spacing distributions provides the characteristic length scale of the NP arrangement.

**Figure 3:**
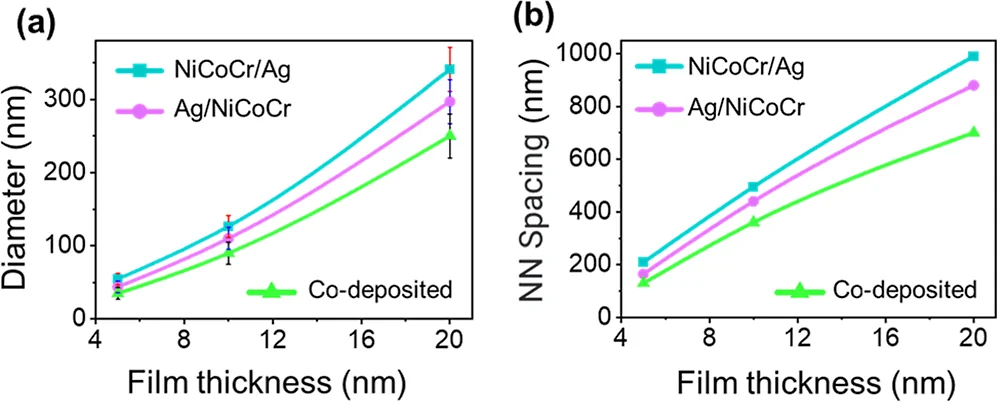
Plots show the (a) diameter and (b) nearest neighbor (NN) spacing of NPs as a function of initial film thickness for NiCoCr/Ag, Ag/NiCoCr, and codeposited configurations, respectively.

To investigate the elemental distribution within the NPs, STEM-EDS characterization was performed on AgNiCoCr NPs fabricated from thin films with thicknesses of ∼5 nm, ∼10 nm, and ∼20 nm across different configurations (NiCoCr/Ag, Ag/NiCoCr, and codeposited) as shown in Figure 4. For NPs derived from thicker films (∼20 nm, Figure 4a), clear phase segregation is observed in all configurations, characterized by an Ag-rich core and a NiCoCr-rich shell. The average NP diameters are 235 ± 25 nm, 215 ± 25 nm, and 200 ± 25 nm for NiCoCr/Ag, Ag/NiCoCr, and codeposited films, respectively. A similar phase-segregated structure persists at intermediate thickness (∼10 nm, Figure 4b), with reduced particle sizes of 110 ± 15 nm and 100 ± 15 nm for NiCoCr/Ag and Ag/NiCoCr, respectively, and ∼95 ± 15 nm for codeposited films. These results indicate that phase segregation remains dominant at larger particle sizes regardless of stacking configuration.

**Figure 4:**
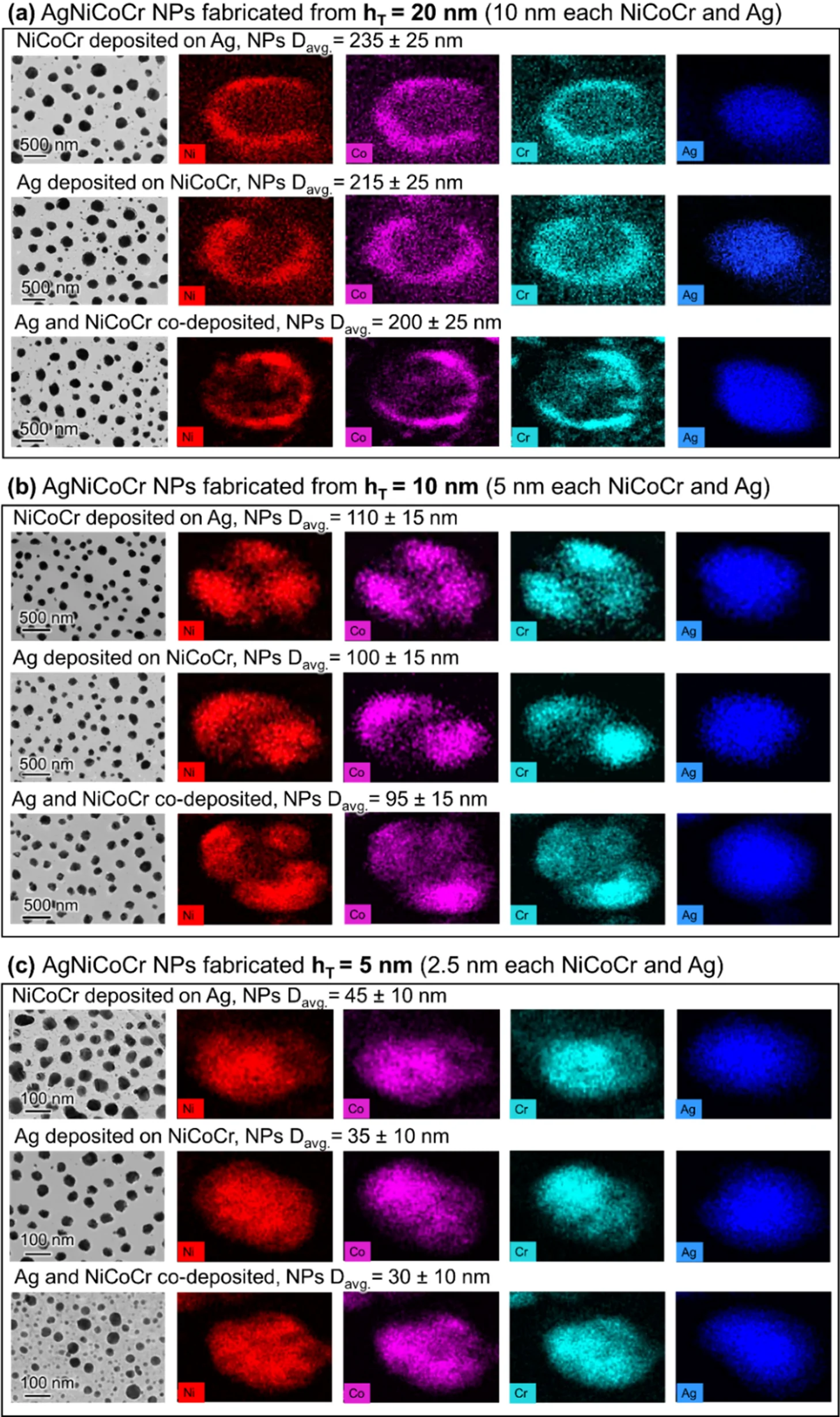
Plan-view TEM images and corresponding STEM–EDS elemental maps (Ni, Co, Cr, and Ag) of AgNiCoCr NPs fabricated via NLiD from thin films with total thicknesses of (a) ∼20 nm, (b) ∼10 nm, and (c) ∼5 nm for NiCoCr/Ag, Ag/NiCoCr, and codeposited NiCoCr–Ag configurations. A transition from phase-segregated structures at larger particle sizes to chemically homogeneous NPs at smaller sizes is observed.

In contrast, NPs fabricated from ultrathin films (∼5 nm, Figure 4c) exhibit uniform elemental distribution, with no evidence of core–shell segregation. The average NP diameters decrease to 45 ± 10 nm (NiCoCr/Ag), 35 ± 10 nm (Ag/NiCoCr), and 30 ± 10 nm (codeposited). Notably, the fraction of chemically homogeneous NPs is higher in the codeposited films (∼95%) compared to the bilayer configurations (∼75%). The average compositions of the NPs, Ag (51 ± 2 at. %), Ni (18 ± 2 at. %), Co (14 ± 2 at. %), and Cr (17 ± 2 at. %), closely match those of the parent thin films in all the scenarios, confirming compositional retention during dewetting.

These results reveal a clear size-dependent transition from phase-segregated to chemically homogeneous NPs, occurring below a critical particle size of ∼45 nm. This transition can be attributed to the competition between diffusion-driven phase separation and rapid solidification during laser processing. In larger NPs, longer liquid lifetimes enable elemental segregation and core–shell formation, whereas in smaller NPs formed from thinner films, rapid quenching suppresses diffusion, resulting in a homogeneous high-entropy solid solution. Overall, these findings demonstrate that reducing the initial film thickness (≤∼5 nm) is critical for achieving chemically homogeneous high-entropy NPs, irrespective of the initial film configuration.

The structural characteristics of the AgNiCoCr nanoparticles were further examined by TEM imaging and selected area electron diffraction (SAED), as shown in Figure 5. Bright-field TEM imaging shows predominantly spherical nanoparticles with an average diameter of ∼22 nm with homogeneous elemental mixing, while the internal diffraction contrast observed within individual particles confirms that the nanoparticles contain well-developed crystalline domains. More importantly, the corresponding SAED pattern exhibits a set of diffraction rings that can be consistently indexed to an FCC structure, indicating that the nanoparticles predominantly crystallize into a single FCC alloy phase rather than separating into distinct elemental or intermetallic phases with different crystal symmetries. Together, the TEM and SAED results suggest that nanosecond laser-induced dewetting enables effective alloying of Ag, Ni, Co, and Cr during rapid melt-state processing, followed by resolidification into crystalline multicomponent nanoparticles with a common FCC structure.

**Figure 5:**
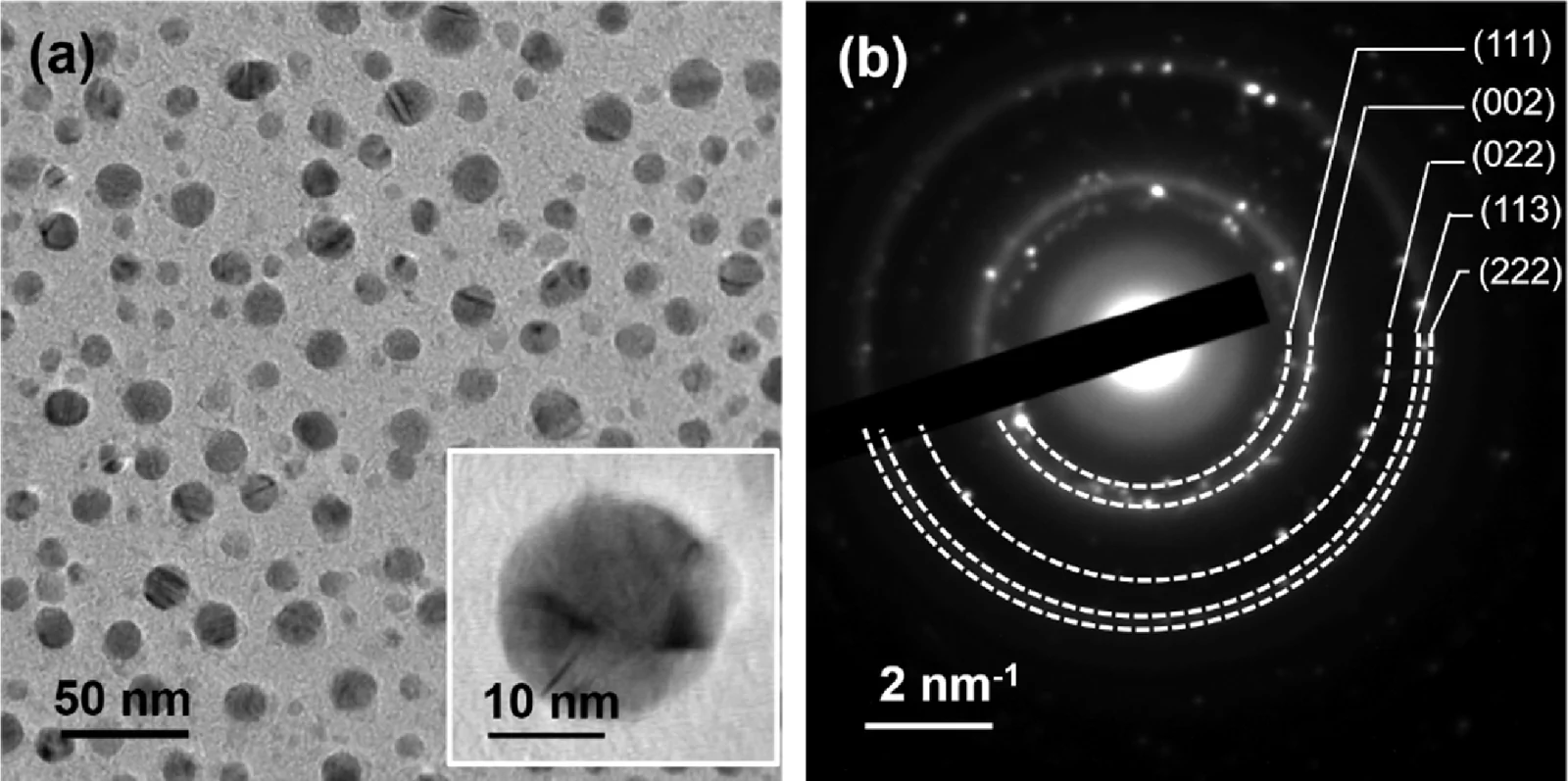
(a) Bright-field TEM image of AgNiCoCr nanoparticles (average diameter ≈22 nm); inset shows a magnified image of an individual nanoparticle. (b) Selected-area electron diffraction (SAED) pattern acquired from the nanoparticle ensemble shown in (a). The TEM micrograph reveals diffraction contrast within the nanoparticles, indicating their crystalline nature. The indexed diffraction rings in the SAED pattern confirm the formation of an FCC structure in the AgNiCoCr nanoparticles.

To elucidate the transition from phase-segregated to chemically homogeneous NPs, it is essential to understand the solidification dynamics during laser-induced dewetting. Figure 6 presents the simulated time-dependent thermal profiles of films with varying thicknesses and configurations (NiCoCr/Ag, Ag/NiCoCr, and codeposited) under identical laser fluence conditions (200 mJ cm^–2^), sufficient to fully melt the films. The simulations reveal that heating and cooling rates differ significantly between NiCoCr and Ag, primarily due to their large contrast in thermal conductivity and melting temperature. Ag, with a much higher thermal conductivity (∼429 W m^–1^ K^–1^), dissipates heat more rapidly than NiCoCr (∼15 W m^–1^ K^–1^), leading to faster cooling. Consequently, the top layer plays a dominant role in governing the overall cooling dynamics of bilayer films. For thicker bilayer films (∼20 nm, Figure 6a), the NiCoCr/Ag configuration exhibits a substantially longer liquid lifetime, remaining molten for ∼28 ns longer than the Ag/NiCoCr configuration. This extended liquid lifetime enhances atomic diffusion and mass transport, promoting elemental segregation and core–shell formation, consistent with the experimental observations in Figure 4a. As the film thickness decreases to ∼10 nm (Figure 6b), the difference in cooling time reduces to ∼12 ns, although segregation remains evident. A further reduction in thickness to ∼5 nm (Figure 6c) significantly minimizes the difference in cooling rates (∼5 ns), resulting in limited diffusion time and partial suppression of phase segregation, leading to predominantly chemically homogeneous NPs, albeit with some residual heterogeneity. This aligns well with the experimental findings.

**Figure 6:**
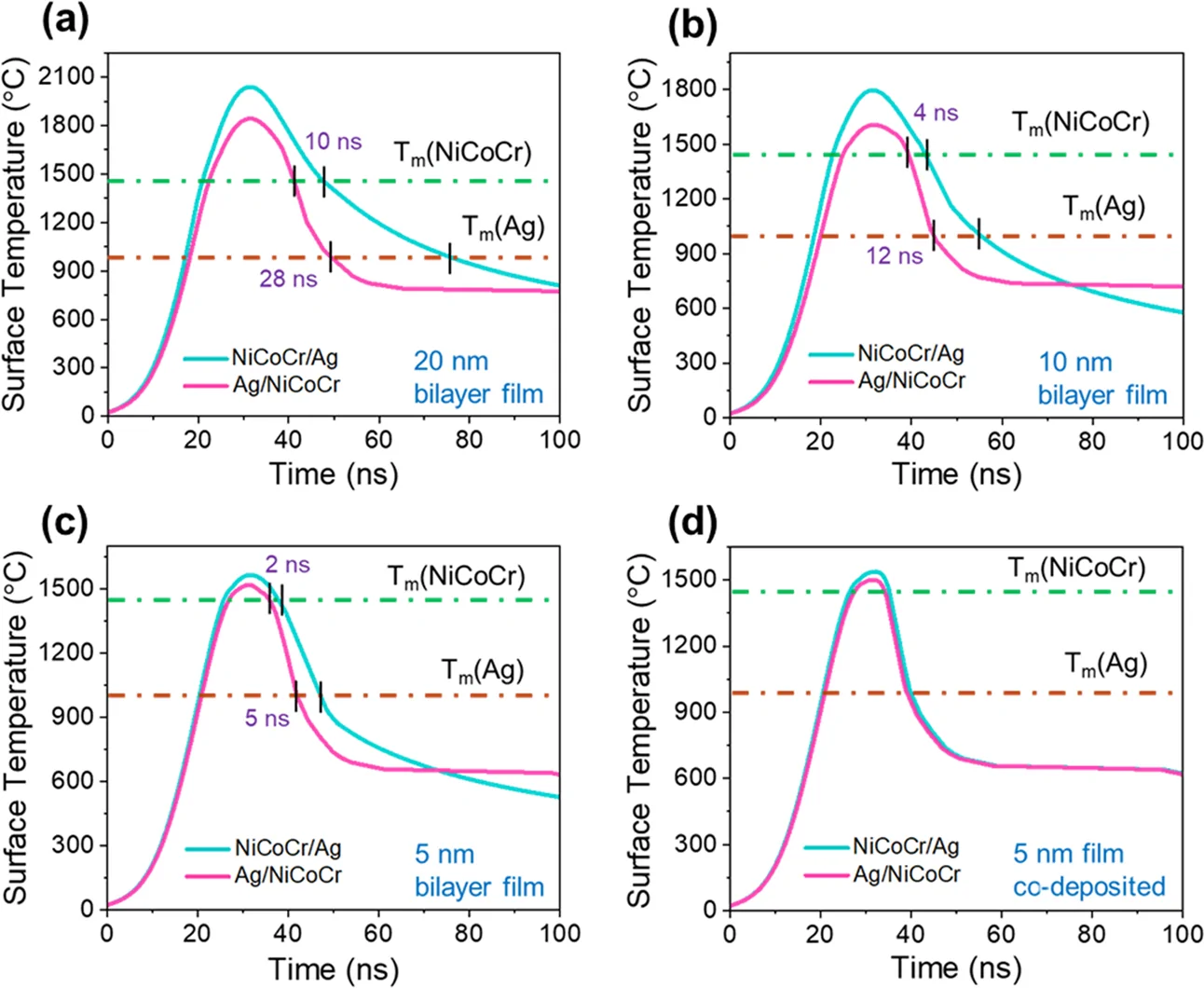
Time-dependent thermal profiles of AgNiCoCr bilayer and codeposited films with thicknesses of (a) ∼20 nm, (b) ∼10 nm, (c) ∼5 nm, and (d) ∼5 nm (codeposited), obtained at a laser fluence of 200 mJ cm^–2^. The dashed lines indicate the melting temperatures of NiCoCr (1417 °C) and Ag (962 °C). The temporal evolution highlights thickness- and configuration-dependent cooling dynamics.

In the case of codeposited films (∼5 nm, Figure 6d), where the effective individual layer thicknesses are extremely small (∼0.5 nm), the thermal profiles of NiCoCr and Ag nearly overlap, indicating negligible differences in cooling behavior. These results demonstrate that the competition between atomic diffusion during the molten state and rapid solidification governs the final chemical structure of the nanoparticles. Thicker films remain molten for longer durations, providing sufficient time for diffusion-driven elemental segregation, whereas thinner or codeposited films undergo rapid quenching that kinetically traps a chemically homogeneous elemental distribution. It should be noted that the present thermal model considers simplified bilayer and codeposited configurations under single-pulse irradiation to capture the essential cooling behavior. As shown in Figure 6, the thermal transport simulations predict nanosecond melt lifetimes that decrease systematically with decreasing film thickness, leading to progressively faster solidification in ultrathin films. Consistent with this trend, the MD simulations (Table 1) predict molten lifetimes ranging from approximately 2–40 ns depending on layer thickness, placing both the simulations and experiments within the same rapid-solidification regime where atomic diffusion directly competes with quenching. Although the detailed thermal histories differ because the experiments involve repeated nanosecond laser pulses whereas the MD simulations employ a simplified thermal cycle, both approaches consistently capture the thickness-dependent transition from chemically homogeneous nanoparticles in ultrathin films to phase-segregated nanoparticles in thicker films.

**Table 1:** Molten Lifetime and Average NP Size from MD Simulations of Ag/NiCoCr Bilayers with 2 Å and 4 Å Ag Interlayers at 2000–3000 K^a^

thickness (nm)	temperature (K)	p Ag	time (τ_liq_) (ns)	average size (L) (nm)	*D*_rel_ (nm^2^ s^–1^)	Σ=DrelτliqL2
2	2000	top	4.38	3.67	2.49	0.81
	2000	bottom	2.00	2.92	2.9	0.68
	2500	top	2.81	3.05	3.18	0.96
	2500	bottom	1.81	2.95	3.02	0.62
	3000	top	2.46	2.47	3.17	1.27
	3000	bottom	2.64	2.90	3.64	1.14
4	2000	top	37.90	8.21	2.1	1.18
	2000	bottom	40.37	6.89	1.96	1.67
	2500	top	27.01	7.83	2.71	1.19
	2500	bottom	31.01	7.43	2.39	1.34
	3000	top	25.94	6.97	2.74	1.46
	3000	bottom	20.49	5.50	2.84	1.92

a Results compare Ag on top vs Ag beneath NiCoCr, showing the effect of stacking order and thickness on NP solidification dynamics.

We further investigated this dewetting phenomenon simulated using MD simulation for an improved mechanistic understanding. Figure 7 demonstrates a representative example of the dewetting phenomenon occurring in bilayer system of 4 Å Ag deposited on 4 Å NiCoCr and equilibrated at 2000 K. While the thicknesses of metallic layers in the simulation are significantly different, they capture the essence of dewetting phenomenon, qualitative mechanistic insights into the early stage atomic processes governing dewetting, such as melting, diffusion, and instability development, in bilayer systems and the understanding of the atomic-scale elemental mixing. The use of femtosecond heating cycles is a commonly adopted approximation to mimic rapid energy deposition, capturing the essential thermal response of the system at the atomic scale. Figure 7 shows the evolution of NPs for a pulse irradiation for 50 ns. At the initial frame, Ag and NiCoCr are intermixed before thermal excitation; upon heating, the system rapidly enters a liquid-like state and undergoes dewetting into bicontinuous structures, followed by resolidification at room temperature. The final snapshots clearly capture the transition from a mixed initial bilayer to phase-separated NPs, reproducing the experimental pathway under pulsed laser irradiation. The MD simulation further facilitates advancing our understanding on the trends of mixing of immiscible systems via developing various analysis metrics from various viewpoints. It should be noted that the apparent agreement between the STEM-EDS maps and the MD simulations must be interpreted in the context of their different spatial resolutions. While the MD simulations resolve atomic-scale compositional fluctuations and predict a slight Ag enrichment confined to the outermost atomic layers, the STEM-EDS maps represent a composition averaged over the electron probe interaction volume and specimen thickness. Consequently, subtle surface enrichment extending over only a few atomic layers is expected to remain below the detection capability of STEM-EDS, whereas larger-scale core–shell segregation is readily resolved experimentally. Therefore, the homogeneous elemental maps observed for the ∼5 nm nanoparticles indicate that long-range phase segregation is effectively suppressed, while not excluding the possibility of weak atomic-scale surface enrichment predicted by the simulations.

**Figure 7:**
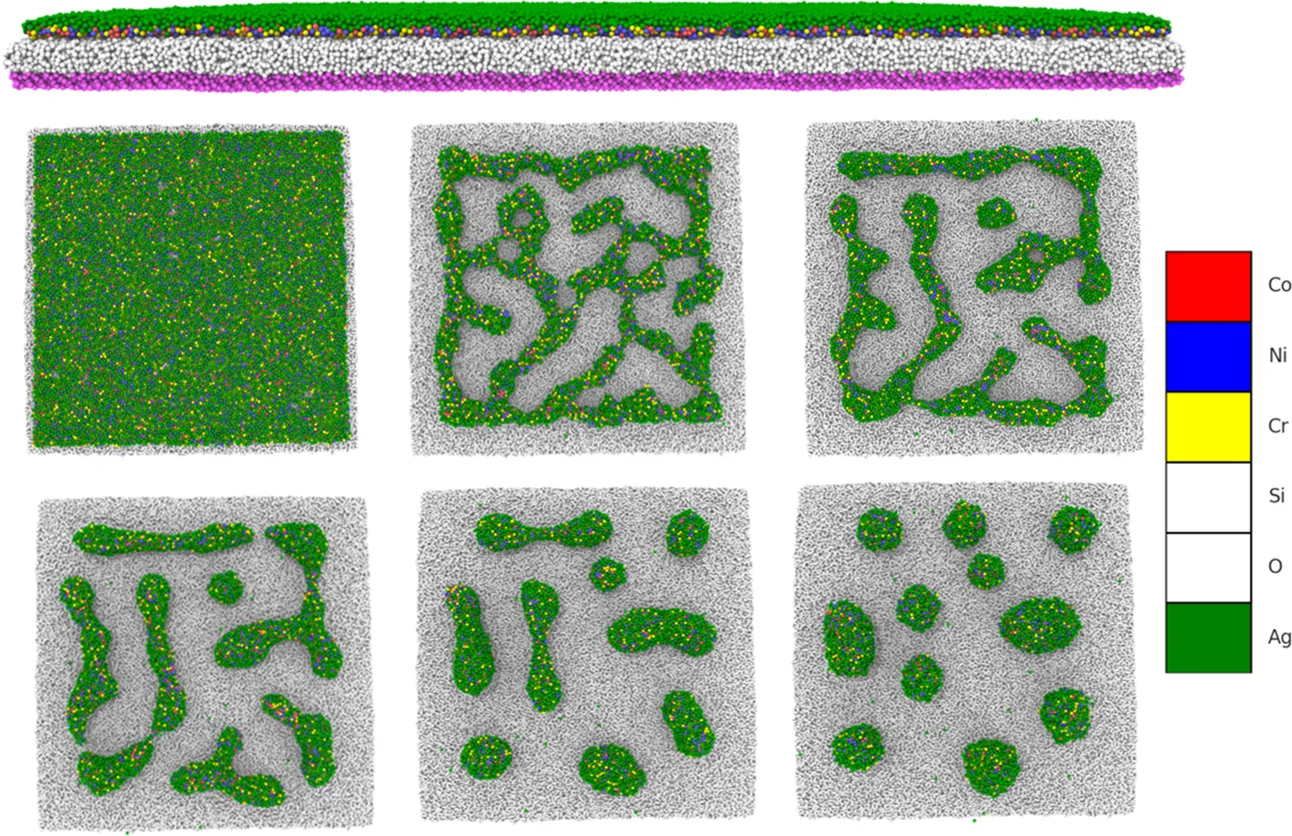
Molecular dynamics simulations of NiCoCr/Ag thin films. The top panel shows a side view of the initial stack with Ag on top of NiCoCr, while the subsequent six panels illustrate the dewetting sequence from ligaments to bicontinuous structures and finally NPs.

To further corroborate the experimental thickness dependence on NP formation, we analyzed segregation dynamics in molecular dynamics trajectories at 3000 K for ultrathin Ag layers of 2–4 Å deposited either on top of or beneath NiCoCr. Here, we define time-resolved segregation metrics, the core–shell index (CSI), surface enrichment factor (*E*), and normalized mean radius (*r*), that describe the likelihood of phase-separation in the form of core–shell or zonal (Janus-like) structures based on the MD simulations (detailed description in Method section 2.4). Figure 8 demonstrates the variation of CSI, *E* and *r* as a function of Ag film thickness which analogically replicates the experimental trends of bilayer configurations with increasing film thickness. The results consistently show that Ag atoms migrate toward the surface during the molten stage, while Ni, Co, and Cr remain clustered in the interior. For Ag initially on top, the contrast between surface and core is strongest at 2 Å (CSI_Ag_ ≈ 0.16, *E*_Ag_ ≈ 1.14; Figure 8) but diminishes toward 4 Å as the larger Ag fraction distributes more evenly. When Ag begins as the underlayer to NiCoCr, segregation is still observed: Ag rapidly migrates outward, with the strongest enrichment at 3 Å (*E*_Ag_ ≈ 1.30, *r̅*_Ag_/*R* ≈ 0.76). In all cases, the equilibrium structure is Ag-enriched surfaces and NiCoCr-rich cores, demonstrating that segregation arises even from ultrathin bilayers once sufficient Ag is present.

**Figure 8:**
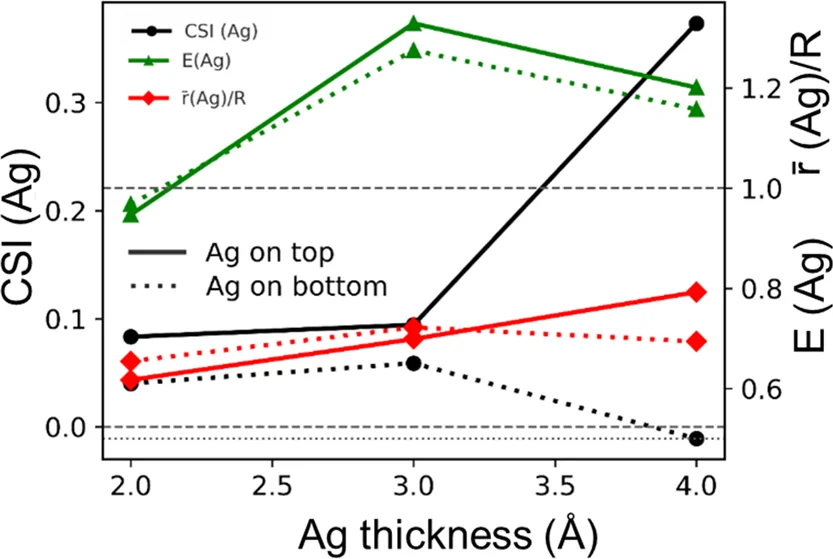
Molecular dynamics (MD) simulation of Ag segregation in AgNiCoCr NPs at 3000 K. The plot shows key segregation metrics, core–shell index (CSI_Ag_), normalized mean radius (*r̅*_Ag_/*R*), and surface enrichment (*E*_Ag_), as a function of initial Ag layer thickness for two configurations: Ag on top of NiCoCr (solid lines) and Ag beneath NiCoCr (dotted lines). The results show that Ag consistently migrates to the NP surface, with the segregation tendency depending on the initial film thickness and configuration.

Beyond the quantitative segregation metrics, the MD trajectories also provide insight into the structural state of the alloy during and after pulsed irradiation. At these ultrathin thicknesses (2–4 Å bilayers) the system undergoes complete melting between 2000–3000 K, followed by extremely rapid quenching. As a result, no persistent long-range crystalline order is recovered upon solidification: common neighbor analysis and polyhedral template matching detect no stable FCC or HCP lattices. Instead, the interior of the NPs exhibits a chemically disordered but compact coordination environment, consistent with a short-range FCC-like packing of Ni, Co, and Cr. Ag, in contrast consistently avoids Ni/Co neighbors and enriches the surface. Thus, the simulations indicate that pulsed laser processing stabilizes NPs not through equilibrium crystallinity, but through a kinetically trapped, chemically disordered structure. This explains why experiments observe compositional homogeneity in ∼5 nm films despite the absence of well-defined crystalline order, the system is frozen into a high-entropy, short-range ordered state rather than relaxing into equilibrium phases.

The MD results shown in Table 1 further provide a kinetic explanation for the thickness-dependent segregation trends that are observed experimentally. Table 1 consolidates the key kinetic parameters, molten lifetime, NP size, and ∑, that move the system across the ∑ = 1 threshold. As explained earlier in the methodology section, segregation number ∑ shows a direct proportionality with diffusivity (*D*_rel_) and molten lifetime (τ_liq_) and inversely proportional to the average NP size (*L*). Σ is introduced as a dimensionless kinetic descriptor derived from molecular dynamics simulations to quantify the competition between atomic diffusion and rapid solidification during nanoparticle formation. Its predictive capability is evaluated by comparing the simulation predictions with the experimentally observed transition from phase-segregated nanoparticles in thicker films to chemically homogeneous nanoparticles in ultrathin films. Since elemental mixing is governed by the combined influence of multiple parameters, it is necessary to consider ∑ holistically to understand the transition between high-entropy mixing and phase segregation at the nanoscale. This atomistic picture dovetails with the experimental finding that solidification rate, facilitated by the difference in film thickness and thermal conductivity of NiCoCr and Ag, governs elemental distribution.

The table shows the molten lifetimes, giving values of 2.0–4.4 ns and corresponding particle radii of ∼2.5–3.7 nm. The resulting segregation number ∑ is mostly less than 1 (ranging 0.63–0.96 at 2000–2500 K), only edging above unity at 3000 K (∑ = 1.14–1.28) due to higher self-diffusion These conditions (lower values of ∑) represent the kinetic trapping regime: solidification outruns Ag diffusion, preserving a more homogeneous alloy state in smaller particles formed in thinner bilayer films. In sharp contrast, Table 1 shows that 4 Å systems remain molten for an order of magnitude longer, with lifetimes of ∼20–40 ns and larger final radii of ∼5.5–8.8 nm. The corresponding ∑ values rise well above unity (∑ = 1.18–1.86), especially when Ag is deposited beneath NiCoCr. These numbers confirm that thicker Ag layers allow sufficient time for diffusion, resulting in pronounced Ag enrichment at the surface and NiCoCr-rich cores. The MD results thus provide quantitative proof of 2 Å interlayers solidify too quickly (2–4 ns) leading to elemental mixing, while 4 Å interlayers linger molten (20–40 ns), enabling segregation which are consistent with the thermal analysis using COMSOL inspite of the scaling difference.

The evidence from Table 1 demonstrates that film thickness directly governs molten lifetime. At 2 Å, the liquid fraction decays within 2–4 ns, leaving no significant window for atomic redistribution. The corresponding ∑ values remain below or near unity, consistent with a kinetically trapped state. This points to a chemically disordered, high-entropy like interior that is frozen in before atomic diffusion pathways can develop. In contrast, at 4 Å, molten lifetimes extend to 20–40 ns, which shifts σ well above unity (1.2–1.8) and provides sufficient time for Ag to diffuse outward. Thus, longer-lived melts at higher thickness enable chemical ordering and segregation, whereas thinner films quench too rapidly, chemically trapping a kinetically stabilized high-entropy state. This mechanistic connection closes the loop between atomistic simulation and experiment: the solidification rate controlled by film thickness and thermal conductivity determines whether AgNiCoCr NPs freeze into a homogeneous distribution (kinetic trapping, ∑ < 1, weak SRO) or evolve into Ag-rich shells and NiCoCr-rich cores (segregation, ∑ > 1, stronger SRO).

## 4 Conclusions

This study demonstrates the successful fabrication of multielement AgNiCoCr NPs via NLiD of bilayer and codeposited thin films. Nanoscale characterization reveals the evolution of dewetting morphologies and the subsequent formation of NPs from NiCoCr/Ag, Ag/NiCoCr, and codeposited configurations. The transformation of molten thin films into droplet-like nanostructures is governed by Rayleigh-like instabilities and thermocapillary-driven mass transport. The NP size and nearest-neighbor spacing are strongly dependent on the film thickness and stacking configuration, reflecting the influence of thermal conductivity and cohesive energy of the constituent elements. STEM-EDS analysis shows that thinner films, codeposited and bilayer films, with thickness ∼ 5 nm yield a chemically homogeneous NPs. In contrast, increasing the film thickness promotes phase separation, resulting in Ag-rich and NiCoCr-rich segregated regions. Molecular dynamics simulations further reveal that the molten lifetime increases significantly with increasing film thickness, shifting the system across a critical kinetic threshold (Σ ≈ 1). Rapid quenching at smaller thicknesses kinetically traps a high-entropy, homogeneous state, whereas longer liquid lifetimes at larger thicknesses enable diffusion-driven elemental segregation, leading to core–shell structures. Overall, the structural evolution of AgNiCoCr NPs is governed by the competition between diffusion and solidification kinetics, which is controlled by the individual layer thickness and thermal properties of NiCoCr and Ag. The strong agreement between microscopy, thermal modeling, and MD simulations establishes film thickness as a master parameter linking kinetics, morphology, and chemical distribution. This work provides both quantitative evidence and mechanistic insight into the formation of multicomponent NPs via laser-induced dewetting, offering a versatile pathway for tailoring NP size and composition for catalytic and energy-related applications.

## Supplementary Material



## Notes

Scanning electron microscopic data: progression of dewetting morphologies in codeposited Ag and NiCoCr films (Figure S1) (PDF)
