# Supplementary material for: AgNiCoCr Nanoparticles: Exploring the Morphological Shift from Elementally Segregated to High Entropy Structures
Source: ACS Appl Nano Mater. 2026 Aug 15;9(34):16258–69. doi: 10.1021/acsanm.6c02061 (PMC13532043; doi:10.1021/acsanm.6c02061)
Supplement: Supplementary file 1 [file an6c02061_si_001.pdf]

Supporting information

AgNiCoCr Nanoparticles: Exploring the Morphological Shift from Elementally Segregated to High Entropy Structures

Soumya Mandal[1], Avik Mahata[2], Annaliese Colwell[3], Vikas Reddy Paduri[1], Nagarajan Anna Ramesh Babu[1], and Ritesh Sachan[1*]

[1] Department of Mechanical and Aerospace Engineering, Oklahoma State University, Stillwater, OK 74078, USA

[2]Department of Mechanical & Electrical Engineering, Merrimack College, North Andover, MA 01845 USA

[3]Department of Materials Science and Engineering, University of Connecticut, Storrs, CT 06269 USA

*Corresponding Email: rsachan@okstate.edu

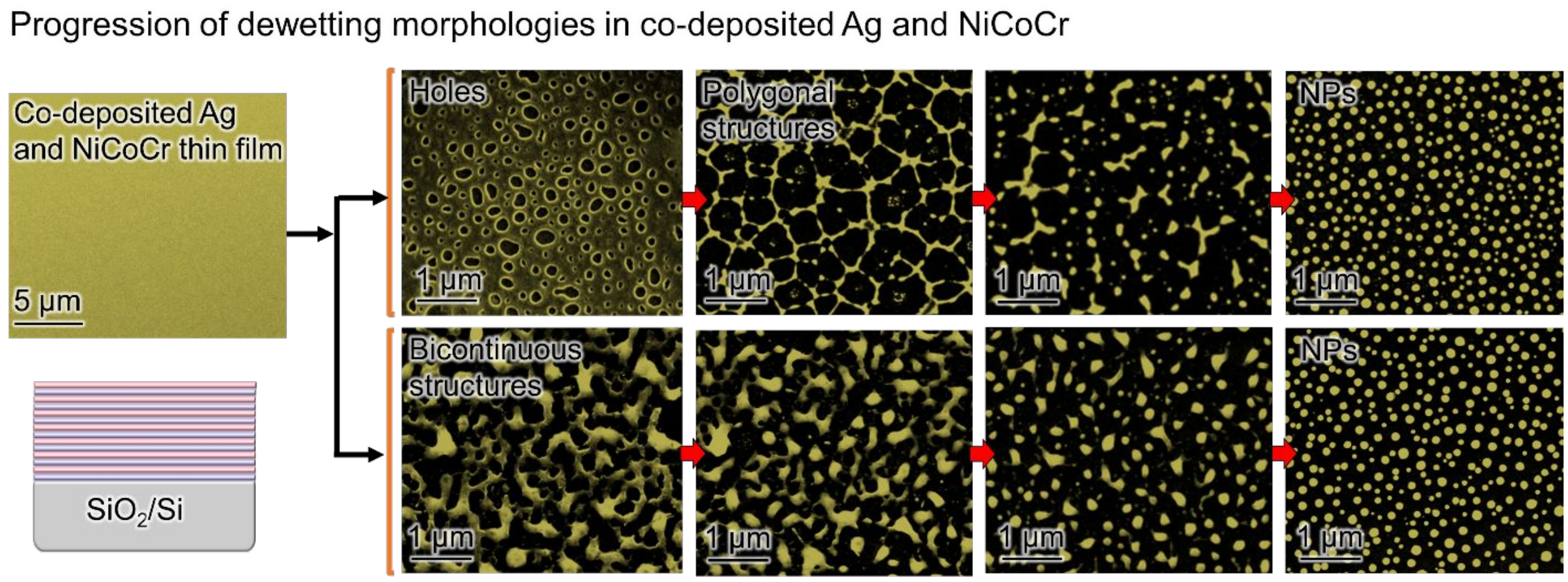

Figure S1. Progress of dewetting morphologies in co-deposited Ag and NiCoCr showing the dewetting pathways through holes and bicontinuous structure formation.
